# Risk and protective factors of student burnout among medical students: a multivariate analysis

**DOI:** 10.1186/s12909-025-06956-8

**Published:** 2025-03-15

**Authors:** Hedvig Kiss, Bettina F. Pikó

**Affiliations:** Department of Behavioral Sciences, Albert Szent-Györgyi Medical School, University of Szeged, Szentháromság utca 5., Szeged, 6720 Hungary

**Keywords:** Burnout, Medical student, Depression, Anxiety, Resilience, Motivation

## Abstract

**Background:**

The demanding educational environment requires sustained motivation and resilience, while the intense psychological stress among medical studies increases the risk of depression, anxiety, and burnout. Student burnout is an escalating issue among medical students worldwide, significantly affecting their career success and overall well-being. Understanding these dynamics is crucial for effective burnout prevention strategies. Therefore, this study aims to explore the role of depression, anxiety, academic motivation and resilience in student burnout in a sample of Hungarian medical students.

**Methods:**

An online survey was conducted among medical students at the University of Szeged, Hungary (*N* = 214; M_age_ = 21.82 years; 73.8% female). The survey first collected demographic information, after which participants completed five scales: the Maslach Burnout Inventory Student Survey, the Beck Depression Inventory Short Form, the Spielberger State-Trait Anxiety Inventory, the Academic Motivation Scale, and the Academic Resilience Scale.

**Results:**

Binary logistic regression for emotional exhaustion identified depression (OR = 0.32, *p* < .001), state anxiety (OR = 0.04, *p* < .01), and amotivation (OR = 0.19, *p* < .01) as significant predictors. For cynicism, the final model incorporated state anxiety (OR = 0.05, *p* < .001), intrinsic motivation through achievement (OR = -0.08, *p* < .05), external regulation (OR = 0.13 *p* < .01), and amotivation (OR = 0.41, *p* < .001). For reduced academic efficacy, depression (OR = 0.15, *p* < .001), alongside achievement (OR = -0.133, *p* < .01) and stimulation (OR = -0.10, *p* < .05) as intrinsic motivations were significant predictors.

**Conclusions:**

This study reveals a significant prevalence of student burnout among medical students, particularly in clinical years, with emotional exhaustion and cynicism being more prominent. Depression and low motivation were strongly associated with higher student burnout, while intrinsic motivation appeared to protect against reduced academic efficacy. These findings underscore the importance of addressing mental health and fostering resilience to mitigate student burnout during medical training.

**Supplementary Information:**

The online version contains supplementary material available at 10.1186/s12909-025-06956-8.

## Background

Burnout, initially introduced by Freudenberger [26] to describe emotional exhaustion among psychiatric staff, was later refined by Maslach and Jackson [42], identifying emotional exhaustion, depersonalization, and reduced personal accomplishment as core dimensions. While their work initially focused on professionals in human services, the concept has since been applied to other groups, including students [24, 43]. Schaufeli et al. [58] adapted Maslach’s model to the academic setting, defining student burnout through the dimensions of emotional exhaustion (emotional depletion due to educational demands), cynicism (an indifferent attitude toward academic activities); and reduced academic efficacy (a diminished perception of personal competence).

The prevalence of student burnout among medical students is a growing concern, with global studies reporting varying rates [9]. Medical students face significant challenges due to the intense demands of their education [13, 47], which can lead to elevated burnout rates. For example, in studies from the US and Europe, burnout rates among medical students range from 7% to 75.2%, with an overall rate of 37.23% [3, 25]. While data for Hungary is limited, similar trends are emerging [1, 30, 32]. The increasing occurrence of burnout throughout the course of medical training has been well-documented, with studies showing significant rises in burnout levels from preclinical to clinical years [20]. Galán et al. [27] observed that each additional year of study correlates with increased burnout levels across all subscales, while Győrffy et al. [30] specifically noted higher cynicism during clinical years, with no significant differences in emotional exhaustion or reduced academic efficacy.

Student burnout among medical students results from an interplay of various risk and protective factors. Its predictors can be categorized into contextual and individual factors, as initially described by Cordes and Dougherty [17]. Contextual factors such as workload, patient care stressors, and difficulties in integrating into healthcare teams contribute to burnout, while individual factors like anxiety, depression, and low resilience increase vulnerability. Expanding on these categories, additional contributors have been described [6], such as interpersonal challenges which can arise from difficulties in establishing relationships due to short rotations,environmental factors, like inadequate study space [23] and poor access to rest,and systemic issues such as academic factors [51] and being perceived as a burden on already overworked healthcare staff [5]. Occupational factors, like a short time to integrate into teams, also contribute to burnout. Situational [69] and sociocultural factors, such as studying and working in unfamiliar contexts, add another layer of stress. In addition to these factors, individual characteristics such as unhealthy lifestyle habits, anxiety, depressive symptoms, lack of motivation, and low resilience also play a significant role in increasing burnout risk [14].

Since most models explain the development of student burnout through the interaction of contextual and individual factors, this study specifically investigates anxiety, depression, academic motivation, and academic resilience. These psychological variables are key components of the risk and protective factors model [38], which distinguishes between factors that exacerbate vulnerability to burnout and those that mitigate it [48]. While previous research has established links between student burnout, depression, and anxiety [37, 44, 53, 60], as well as the protective effects of motivation and resilience [29], the nuanced interplay of these variables in the context of medical training remains underexplored. For instance, the reciprocal relationship between student burnout and depression is well-researched [12, 56, 63], but the role of anxiety as a potential stable trait contributing to emotional exhaustion warrants further investigation [59]. While it is known that medical students with positive self-perception, strong motivation for studying [8, 68], and adequate level of resilience are less likely to develop burnout [41, 50] as these factors help them manage the emotional demands of medical training [21, 35], it remains unclear how these protective factors interact with risk factors across different stages of medical education.

Addressing these gaps, this study focuses on medical students, whose unique academic stressors amplify the need for targeted research. Previous findings underscore the importance of understanding student burnout not only for improving student well-being but also for ensuring sustainable healthcare systems [10, 22, 66]. By examining anxiety, depression, academic motivation, and academic resilience through the lens of the risk and protective factors model, this study provides new insights into the mechanisms underlying student burnout among medical students. Specifically, the study has one primary objective and two secondary objectives: 1) To examine the role of psychological (risk and protective) factors such as depression, anxiety, academic motivation, and academic resilience in relation to burnout (primary),2) To analyze how burnout dimensions differ across the stages of medical training (preclinical vs. clinical) (secondary); and 3) To explore the overall occurrence of student burnout among medical students (secondary).

## Methods

### Participants

This cross-sectional pilot study surveyed medical students in the spring semester of the academic year 2023/2024 at the Albert Szent-Györgyi Medical School, University of Szeged, Hungary. The target population’s distribution across academic years and biological sex was obtained from preliminary data provided by the students’ secretariat. Based on this information, the authors expected a female surplus and more participants from the preclinical years (years 1, 2, and 3). To promote inclusivity and broad representation, the study aimed to reach as many medical students as possible within the Hungarian program. Therefore, all medical students (with active student status) in the Hungarian program were informed about the study. The total population included 490 males (38%) and 791 females (62%), from which the authors received responses from 56 males (11.43% of the total male population) and 158 females (19.97% of the total female population). The response rates varied across academic years, with Year 1 having the highest response rate of 23.2%, followed by Year 3 with 15.7%, Year 2 with 15.5%, Year 4 with 18.7%, Year 5 with 15.2%, and Year 6 with the lowest response rate of 9.6%.

Participation in the study was entirely voluntary, and students were free to decide whether to take part without facing any negative consequences for non-participation. To encourage participation, students had the option to earn points for completing the questionnaire, as arranged beforehand by a student organization within the faculty. These points could be used for various benefits when applying for international exchange programs. To claim these points, students were required to provide their name, email address, and student identification number, which were handled exclusively by the student organization’s administrator. The research dataset was anonymized before analysis to ensure the confidentiality of participants’ information.

### Data collection

Online data collection was conducted using an interactive survey created and hosted on *Typeform*. The hyperlink to the survey was distributed via multiple channels to maximize participation. Specifically, the link was shared in social media groups of each academic year, on different educational platforms of the University, and via an official email sent by the students’ secretariat to all medical students. Additionally, the survey link was posted on the faculty’s social media pages.

After reading detailed information about the purpose and method of data collection, participants gave informed consent by clicking “I consent”. Students were then informed about the opportunity to earn points for completing the questionnaire. Those who were not interested in earning points and, therefore, did not provide personal details, were immediately directed to the demographic questions. If they wished to receive these points, they could provide the necessary personal details before proceeding further. These personal data were stored separately and not linked to the anonymized dataset used for analysis.

### Measuring instruments

Following these previous steps, participants answered demographic questions, after which validated questionnaires were administered to assess five psychological variables: student burnout, depression, anxiety, academic motivation, and academic resilience.

#### Student burnout

Student burnout was evaluated using the Hungarian validated version [32] of the Maslach Burnout Inventory Student Survey (MBI-SS) [58]. This self-administered questionnaire consists of 15 items across three dimensions: emotional exhaustion was measured with five items (e.g., “I feel emotionally drained by my studies”), cynicism with four items (e.g., “I have become more cynical about the potential usefulness of my studies”), and academic efficacy with six items (e.g., “I have learned many interesting things during the course of my studies”). Participants rated their responses on a 7-point frequency scale from 0 (never) to 6 (always). High emotional exhaustion and cynicism scores, along with low academic efficacy scores (reverse-scored items) indicated burnout. The Cronbach’s alpha coefficients were 0.88 for emotional exhaustion, 0.88 for cynicism, and 0.80 for academic efficacy, which were considered adequate based on previous Hungarian study findings (0.80, 0.85, and 0.82, respectively) [30]. The Hungarian version of the MBI-SS, as published by [32], is accessible and has been previously utilized in similar study of Hungarian medical students [30].

To assess student burnout levels, we adopted the dichotomization method utilized by Győrffy et al. [30], who categorized burnout into two levels (low/moderate and severe) based on scores that were at least half a standard deviation above or below the mean. This approach, though not without its limitations, was chosen to capture the full spectrum of burnout levels, ranging from medium to high risk, moreover, it may include students with average scores in the at-risk population, necessitating careful interpretation based on existing literature. The aggregation of moderate and high levels of burnout was based on the theoretical understanding that burnout represents a continuum of risk, where even moderate levels of emotional exhaustion, cynicism, or reduced academic efficacy may significantly impair students’ mental health and academic performance. In this study, the same criteria were applied to classify burnout, using cut-off points corresponding to the 66th percentile for emotional exhaustion and cynicism, and the 33rd percentile for academic efficacy, since this latter scale is inverted (that is, the lower the score, the greater the chance of characterizing burnout). This resulted in the following thresholds: medium-level burnout: 13.32–20.52 for emotional exhaustion, 4.83–11.99 for cynicism, and 21.76–27.82 for reduced academic efficacy; high-level burnout: > 20.52 for emotional exhaustion, > 11.99 for cynicism, and < 21.76 for academic efficacy.

To contextualize these findings, we compared both the cut-off values and mean scores of our sample with those reported in other Hungarian and international studies. Costa et al. [18] defined high level of student burnout with thresholds of emotional exhaustion (> 14), cynicism (> 6), and reduced academic efficacy (< 23). In terms of mean scores, their sample exhibited higher averages: emotional exhaustion (16.3 ± 6.5), cynicism (7.4 ± 5.7), and reduced academic efficacy (27.9 ± 5.6). Similarly, [32] reported mean scores for Hungarian medical students as follows: emotional exhaustion (10.52 ± 5.95), cynicism (6.75 ± 4.98), and academic efficacy (13.43 ± 6.06). However, in their study, the academic efficacy scale was not reverse-coded, which accounts for the discrepancy in the range of values compared to our findings. Their classification of middle-range burnout included emotional exhaustion (8–14), cynicism (5–10), and academic inefficacy (1–17). Compared to the international and Hungarian averages, the current sample exhibited slightly higher thresholds for emotional exhaustion and cynicism, while the academic efficacy values were comparable after accounting for the reverse scoring methodology. Our study’s cut-off values and mean scores, detailed in Table 1, align with the ranges reported in these studies while also reflecting methodological and contextual differences (variations in sample composition, cultural factors, and the reverse coding of the academic efficacy scale) – such differences underscore the importance of interpreting burnout classifications within their respective frameworks.

**Table 1:** Cut-off values for burnout levels

Burnout dimensions	Lower cut-off of the medium level	Upper cut-off of the medium level
Emotional exhaustion	13.32	20.52
Cynicism	4.83	11.99
Reduced academic efficacy	21.76	27.82

#### Depressive symptoms

Having good reliability and validity across different populations, depression was assessed using the Hungarian shortened version of the Beck Depression Inventory (BDI) [57], originally developed by Beck and Beck in 1972 [4]. This instrument comprises 9 items evaluating various aspects of depression, such as fatigue (“I am too tired to do anything”) and loss of interest (“I have lost all of my interest in other people”). Participants rated each item based on their experiences over the past month, using a scale from 1 (not at all like me) to 4 (very much like me). Higher total scores indicated more severe depressive symptoms: 0–9 indicated no depressive symptoms, 10–18 suggested mild symptoms, 19–25 indicated moderate severity, while scores above 25 reflected severe depressive symptoms. The Hungarian version’s reliability was established with a Cronbach’s alpha of 0.83 [57],in this study, it was 0.87.

#### State anxiety

The level of state anxiety was measured using the Hungarian version of the State-Trait Anxiety Inventory for Adults (STAI-AD) [61], developed by Spielberger et al. [62]. This inventory assesses both state (transient) and trait (enduring) anxiety, though only the state anxiety subscale was used in this study. The STAI-AD consists of 40 items, 20 for each subscale, with participants rating their feeling on a 4-point Likert scale from 1 (almost never) to 4 (almost always). Ten of the 20 state anxiety items are reverse scored (e.g. “I feel secure”), and higher total scores indicate greater anxiety. Scores range from 20 to 80, with Hungarian norms indicating male averages of 38.40 (SD = 10.66) and female averages of 42.64 (SD = 10.79) [39]. The reliability of the state anxiety subscale in this study was 0.96, aligning with previous Hungarian findings of 0.95 [45].

#### Academic motivation

The Hungarian version of the Academic Motivation Scale (AMS) [64] adapted from Vallerand et al. [65], was used to measure academic motivation, applying the self-determination theory [19]. This scale evaluates three dimensions across seven subscales: intrinsic motivation by knowledge (e.g. “I experience pleasure and satisfaction while learning new things”, accomplishment (e.g. “For the satisfaction I feel when I am in the process of accomplishing difficult academic activities”, and stimulation (e.g. “For the pleasure that I experience when I am taken by discussions with interesting teachers”,extrinsic motivation by identified regulation (e.g. “Because I think that a high-school education will help me better prepare for the career I have chosen”, introjected regulation (e.g. “To prove to myself that I am capable of completing my high-school degree”, and external regulation (e.g. “In order to obtain a more prestigious job later on”; and finally, amotivation (e.g. “I once had good reasons for going to school; however, now I wonder whether I should continue”. Participants rated items on a Likert scale from 1 (strongly disagree to 7 (strongly agree, indicating motivation levels. The reliability coefficients for this sample were 0.87 for knowledge, 0.83 for accomplishment, 0.81 for stimulation, 0.70 for identified regulation, 0.81 for introjected regulation, 0.77 for external regulation, and 0.83 for amotivation, similar to those found in previous research [36]: 0.78, 0.79, 0.71, 0.79, 0.70, 0.82, and 0.80, respectively.

#### Academic resilience

The Hungarian adaptation [40] of the Academic Resilience Scale (ARS-30) [16] assessed academic resilience. This 30-item scale measures resilience across dimensions such as perseverance, reflective help-seeking, and negative emotional responses. After reading a vignette describing an academic challenge, participants rated their agreement with statements like “I would use the feedback to improve my work” (perseverance), “I would seek help from my tutors” (help-seeking), and “I would feel like everything was ruined and was going wrong” (negative effects on emotional response) on a five-point Likert scale from 1 (likely) to 5 (unlikely). Positively phrased items were reverse-scored, with higher scores indicating greater resilience. The global score was the sum of all 30 items (range: 30 to 150). Internal reliability was adequate: perseverance (α = 0.76), help-seeking (α = 0.66), and negative effects (α = 0.84), while the entire questionnaire showed excellent reliability (α = 0.87), consistent with previous findings: 0.79, 0.76, 0.75, and 0.82, respectively [40].

### Statistical analysis

All analyses were conducted using SPSS version 25.0, with statistical significance set at or below 0.05. There were no missing data, as all questions were set as mandatory, preventing participants from proceeding without completing each item. Descriptive statistics (frequencies, means, standard deviations, ranges, min–max values) were first used to summarize the data. Next, independent sample t-tests and chi-square tests were applied to compare categorical variables like gender and academic years. To explore the relationship between student burnout and the psychological variables of interest, a multivariate analysis using binary logistic regression was performed.

Power analyses were conducted using G*Power to determine the required sample sizes. For MANOVA, a sample size of 54 was needed to achieve 80% power with an effect size of 0.35, while for binary logistic regression, a sample size of 59 was required for 80% power. With 214 participants, the sample size exceeds this threshold, ensuring sufficient power for both analyses.

Binary logistic regression is a method used to analyze the relationship between a binary dependent variable and one or more independent variables. It is especially helpful when the outcome of interest is binary, such as the presence or absence of a condition. This method estimates the probability of an event based on the independent variables, providing odds ratios to quantify the strength of associations. For example, an odds ratio above 1 suggests that an increase in the independent variable raises the odds of the outcome occurring, while an odds ratio below 1 indicates a negative association. Logistic regression uses a logistic function to ensure that the predicted probabilities range between 0 and 1. Several diagnostic statistics, such as the Hosmer–Lemeshow test and pseudo-R-squared values, help assess the model’s goodness-of-fit. In the stepwise forward regression approach, variables are entered into the model sequentially based on their statistical significance (*p* < 0.05). In the initial step, the most significant predictor is included, and in subsequent steps, additional variables are added if their *p*-value is below the threshold. This automated, data-driven process ensures that only the most relevant predictors are included in the final model. Unlike hierarchical regression, the order of entry is determined by statistical criteria rather than predefined by the researchers. Therefore, in this study, binary logistic regression with stepwise approach was employed to identify the most influential psychological factors, including depression, state anxiety, academic motivation and academic resilience, contributing to the likelihood of student burnout – dichotomized as low/medium and high – while producing odds ratios for each variable. The way of categorizing burnout followed a similar approach as in a previous Hungarian study [30], where burnout was split into two categories: 1. category: low and 2. category: medium and high. Categorical variables, such as academic year and biological sex, were also included in the analysis and coded accordingly: 1 = preclinical years (reference), 2 = clinical years,1 = male (reference), 2 = female. This analysis aimed to identify psychological predictors of student burnout among medical students.

## Results

### Demographic data

The study sample (Additional file 1) consisted of 214 medical students, predominantly females (73.8%, *n* = 158), with males representing 26.2% (*n* = 56). Participants had an average age of 21.82 years (SD = 2.63, range: 18–35) and a median age of 21 years. Participants were distributed across different academic years, with the highest representation in the first year (25.7%, *n* = 55), followed by the second year (21.0%, *n* = 45), third year (17.3%, *n* = 37), fourth year (18.2%, *n* = 39), fifth year (10.7%, *n* = 23), and the least in the sixth year (7.0%, *n* = 15).

### Independent variables

Descriptive statistics for the independent variables – state anxiety, depression, intrinsic and extrinsic motivation, and resilience – are provided in Table 2. The mean score of depression was 15.48 ± 5.23 (range: 9–30), which reflects mild levels of depressive symptoms, according to the Beck Depression Inventory. The state anxiety mean score was 45.93 ± 13.30 (range: 21–80), indicating moderate to high levels of anxiety among the participants, exceeding Hungarian normative mean scores.

**Table 2:** Means, standard deviations, and ranges of independent variables

Variable	Mean ± SD	Range (min–max)
Depression	15.48 ± 5.23	21 (9–30)
State anxiety	45.93 ± 13.30	59 (21–80)
IM – knowledge	22.66 ± 4.79	23 (5–28)
IM – accomplishment	18.27 ± 5.79	24 (4–28)
IM – stimulation	17.50 ± 5.46	24 (4–28)
EM – identified regulation	23.38 ± 3.78	20 (8–28)
EM – introjected regulation	18.27 ± 6.47	24 (4–28)
EM – external regulation	21.23 ± 5.26	24 (4–28)
Amotivation	7.62 ± 4.74	22 (4–26)
RES – perseverance	52.71 ± 6.58	40 (26–66)
RES – help-seeking	28.52 ± 4.91	25 (15–40)
RES – negative effects	20.63 ± 5.94	28 (7–35)

*IM* intrinsic motivation, *EM* extrinsic motivation, *RES* resilience

Academic motivation was assessed in three main dimensions: intrinsic motivation, extrinsic motivation, and amotivation. Intrinsic motivation showed moderate levels, with the highest mean score observed for knowledge-oriented motivation (22.66 ± 4.79, range: 5–28). Accomplishment-oriented motivation and stimulation-oriented motivation had slightly lower means of 18.27 ± 5.79 (range: 4–28) and 17.50 ± 5.46 (range: 4–28), respectively. Among the extrinsic motivation dimensions, identified regulation had the highest mean score, 23.38 ± 3.78 (range: 8–28), suggesting a strong alignment of external goals with personal values. External regulation also showed relatively high levels, with a mean score of 21.23 ± 5.26 (range: 4–28), while introjected regulation had a mean score of 18.27 ± 6.47 (range: 4–28), indicating internal pressures, such as guilt or obligation, were moderately influential. Amotivation was found to be low, with a mean of 7.62 ± 4.74 (range: 4–26), indicating that participants generally exhibit minimal lack of motivation toward their studies.

The dimensions of resilience demonstrated variability. The highest mean score was observed in the persistence dimension (52.71 ± 6.58, range: 26–66), suggesting that participants generally display strong adaptability in challenging situations. The help-seeking dimension had a mean score of 28.52 ± 4.91 (range: 15–40), indicating that participants are generally willing to seek help when needed. Finally, the negative effects dimension had a mean of 20.63 ± 5.94 (range: 7–35), suggesting moderate susceptibility to external stressors or negative influences within the sample.

### Student burnout

Student burnout levels were categorized based on previously established cut-off points, as detailed in the *Methods* section. This approach was chosen to capture the full spectrum of moderate to high burnout risk acknowledging that even moderate levels of burnout dimensions can have a significant impact on students’ well-being. Table 3 shows the prevalence of burnout across its three dimensions. Emotional exhaustion was reported at low levels by 35.0% of participants, while 65.0% exhibited medium or high levels. Cynicism was low for 38.3% but medium or high for 61.7%. Reduced academic efficacy was predominantly low (74.8%), with 25.2% experiencing medium or high levels.

**Table 3:** Burnout prevalence in the sample (*N* = 214)

Burnout dimensions	Low		Medium and high	
	%	n	%	n
Emotional exhaustion	35.0	75	65.0	139
Cynicism	38.3	82	61.7	132
Reduced academic efficacy	74.8	160	25.2	54

The Chi-Square test results presented in Table 4 revealed significant differences in burnout prevalence between preclinical and clinical year students for emotional exhaustion and cynicism. Specifically, emotional exhaustion was identified in 60.6% of preclinical students and 72.7% of clinical students (χ^2^ = 3.19, *p* = 0.050), indicating that clinical students may experience slightly higher levels of emotional exhaustion compared to their preclinical counterparts. Similarly, cynicism was observed in 54.0% of preclinical students compared to 75.3% of clinical students (χ^2^ = 9.48, *p* = 0.002), showing that cynicism is notably more prevalent among clinical students. In contrast, reduced academic efficacy was reported by 25.5% of preclinical students and 24.7% of clinical students (χ^2^ = 0.020, *p* = 0.888), indicating no significant difference between the groups. This suggests that academic efficacy does not vary considerably between the stages of training.

**Table 4:** Burnout prevalence in different stages of training

Burnout dimensions	Preclinical years		Clinical years		χ^2^ value	df	*p*
	%	n	%	n			
Emotional exhaustion	60.6	83	72.7	56	3.19	1	.050
Cynicism	54.0	74	75.3	58	9.48	1	.002
Reduced academic efficacy	25.5	35	24.7	19	0.02	1	.888
Total	100	137	100	77			

Percentage values are based on the number of participants in each group. *p*-values are from the Chi-Square Test

As described in Table 5, multivariate analysis of variance (MANOVA) revealed significant differences among academic year groups regarding burnout levels across all three dimensions. Multivariate tests indicated an overall statistically significant effect of stage of training on burnout (Pillai’s Trace = 0.33, *p* < 0.001). This result underscores the influence of the academic year on students’ experiences of burnout, likely reflecting the increasing demands and stressors as students progress through their training.

**Table 5:** Multivariate Analysis of Variance (MANOVA) results for burnout dimensions by stage of training

Effect	Test statistic	Value	F	Hypothesis df	Error df	*p*-value
Stage of training	Pillai’s Trace	0.33	5.14	15	624	< .001
	Wilks' Lambda	0.69	5.58	15	569.08	< .001
	Hotelling's Trace	0.44	5.99	15	614	< .001
	Roy's Largest Root	0.39	16.11	5	208	< .001

The table presents multivariate and univariate tests of between-subjects effects for MANOVA analyzing emotional exhaustion, cynicism, and reduced academic efficacy

Post-hoc Bonferroni tests further identified distinct differences in burnout dimensions. For emotional exhaustion, significant differences were observed between Year 1 and Year 3 (MD = −5.07, *p* = 0.010) and between Year 1 and Year 5 (MD = −7.26, *p* = 0.001). These findings suggest that emotional exhaustion intensifies particularly during the clinical years. For cynicism, significant differences were found between Year 1 and Years 2 (MD = −4.0101, *p* = 0.029), 3 (MD = −4.28, *p* = 0.027), 4 (MD = −5.85, *p* < 0.001), 5 (MD = −11.23, *p* < 0.001), and 6 (MD = −8.66, *p* < 0.001), indicating a progressive increase in cynicism throughout medical training. These significant results are detailed in Table 6, with all post-hoc comparisons listed in Additional file 2. However, it is noteworthy that no significant differences were found in reduced academic efficacy across different year groups, suggesting that this dimension of student burnout may not be directly tied to the stage of training but could instead stem from other factors. Similarly, no significant differences in any burnout dimensions were observed between biological genders, implying that gender may not be a critical determinant of burnout in this sample (data not shown in the table). Levene’s test indicated uneven error variances for cynicism (Levene's Statistic = 4.987, *p* < 0.001), suggesting caution in interpreting these particular results due to potential variance discrepancies.

**Table 6:** Post-hoc tests results for burnout dimensions by stage of training (Bonferroni correction)

Dependent variable	Academic year comparison	Mean difference	Std. error	*p*-value	95% Confidence Interval
Emotional exhaustion	1–3	−5.07^a^	1.48	.010	−9.43 to −0.70
	1–5	−7.26^a^	1.72	.001	−12.36 to −2.16
Cynicism	1–2	−4.01^a^	1.28	.029	−7.80 to −0.22
	1–3	−4.28^a^	1.35	.027	−8.28 to −0.26
	1–4	−5.85^a^	1.33	.000	−9.80 to −1.90
	1–5	−11.23^a^	1.58	.000	−15.92 to −6.54
	1–6	−8.66^a^	1.85	.000	−14.15 to −3.16

Only significant results are shown. The table displays all significant mean differences based on post-hoc tests after MANOVA analysis using Bonferroni correction. Full post-hoc results can be found in the supplementary materials

^a^The mean difference is significant at the 0.05 level

### Connection between psychological health and student burnout indicated (multivariate analysis)

First, as seen in Table 7, binary logistic regression was conducted to examine the relationship between depressive symptoms, anxiety levels, academic motivation and resilience, and the likelihood of emotional exhaustion. Model fit improved across successive steps as evidenced by decreasing −2 Log likelihood values and increasing Cox & Snell R^2^ and Nagelkerke *R*^*2*^ values (not shown in table). In Step 1, depression showed a statistically significant association with emotional exhaustion (B = 0.44, Wald = 43.60, *p* < 0.001). The odds ratio (OR = 1.55, 95% CI = 1.36–1.76) indicates that for each one-unit increase in depression, the likelihood of emotional exhaustion increases by 55%. In practical terms, this means that students who experience higher levels of depressive symptoms are significantly more likely to experience emotional exhaustion. Step 2 introduced amotivation, which also showed a strong association (B = 0.20, Wald = 7.88, *p* < 0.01). The odds ratio (OR = 1.22, 95% CI = 1.06–1.41) suggests that a one-unit increase in amotivation increases the odds of emotional exhaustion by 22% which implies that students with lower motivation to engage in academic tasks are at a greater risk of emotional exhaustion. Step 3 added state anxiety, which exhibited a marginal association (B = 0.04, Wald = 4.14, *p* < 0.05), while the odds ratio (OR = 1.04, 95% CI = 1.00–1.09) indicates that for each one-unit increase in state anxiety, the likelihood of emotional exhaustion increases by 4%. This shows a small, but significant, contribution of anxiety to emotional exhaustion in students. Multicollinearity was also assessed by examining the tolerance and Variance Inflation Factor (VIF) values for all predictors. The tolerance values were all above 0.2 and the VIF values were all below the critical threshold of 5, indicating no significant multicollinearity issues in the model. The overall model fit was assessed using the Hosmer–Lemeshow test, indicating good fit across all steps (Step 1: χ2 = 2.43, df = 7, *p* = 0.93; Step 2: χ2 = 4.49, df = 8, *p* = 0.811; Step 3: χ2 = 10.59, df = 8, *p* = 0.226).

**Table 7:** Binary logistic regression analysis of emotional exhaustion dimension of burnout

Step	Chi-square (H–L test); (*p*-value)	Variable	B	SE	Wald	df	OR	95% CI
1	2.43 (.932)	Depression	0.44	0.07	43.60	1	1.55***	1.36–1.76
		Constant	−5.45	0.87	39.07	1	0.00***	
2	4.49 (.811)	Depression	0.40	0.07	32.81	1	1.49***	1.30–1.70
		Amotivation	0.20	0.07	7.87	1	1.22**	1.06–1.41
		Constant	−6.11	0.97	39.60	1	0.00***	
3	10.59 (.226)	Depression	0.32	0.08	17.67	1	1.38***	1.19–1.60
		State anxiety	0.04	0.02	4.14	1	1.04**	1.00–1.09
		Amotivation	0.19	0.07	7.33	1	1.21**	1.06–1.40
		Constant	−6.83	1.06	41.29	1	0.00***	

The odds ratios characterizing the relationship between each independent variable and the dependent variable are derived from multiple logistic regression analyses conducted in a stepwise manner

*H–L test* Hosmer and Lemeshow test, *OR* odds ratio, *SE* standard error, *df* degree of freedom, *CI* confidence interval

^**^p < .01

^***^p < .001

Second, the relationship between the aforementioned variables and cynicism as the dependent variable was examined (as displayed in Table 8). Similarly to binary logistic regression of emotional exhaustion, model fit improved across successive steps (not shown in the table). In Step 1, amotivation demonstrated a significant association with cynicism (B = 0.55, Wald = 32.07, *p* < 0.001), where the odds ratio (OR = 1.73, 95% CI = 1.43–2.09) suggested that for each one-unit increase in amotivation, the odds of cynicism increased by 73%. That is, students who lack motivation are significantly more likely to exhibit feelings of cynicism. Step 2 added external regulation, which also showed a significant association (B = 0.10, Wald = 9.13, *p* = 0.003), along with amotivation (B = 0.54, Wald = 29.61, *p* < 0.001). The odds ratio for external regulation (OR = 1.11, 95% CI = 1.04–1.19) indicates a 11% increase in the odds of cynicism for each one-unit increase in external regulation. Step 3 incorporated state anxiety, revealing a statistically significant association (B = 0.04, Wald = 7.81, *p* = 0.005), in addition to external regulation (B = 0.10, Wald = 8.22, *p* < 0.01), and amotivation (B = 0.471, Wald = 22.13, *p* < 0.001). The odds ratio (OR = 1.05, 95% CI = 1.01–1.08) shows that for each one-unit increase in state anxiety, the likelihood of cynicism increases by 5%. Finally, Step 4 included state anxiety (B = 0.05, Wald = 8.22, *p* < 0.01), along with achievement (intrinsic motivation), which also demonstrated a significant association (B = −0.08, Wald = 5.17, *p* < 0.05), external regulation (B = 0.13, Wald = 11.18, *p* < 0.001), and amotivation (B = 0.41, Wald = 17.12, *p* < 0.001). The odds ratio for achievement (OR = 0.92, 95% CI = 0.86–0.99) suggests that for each one-unit increase in achievement motivation, the odds of cynicism decrease by 8%. This suggests that students who find intrinsic motivation through achievement are less likely to feel cynical. For the cynicism dimension of burnout, the regression analysis also revealed no significant multicollinearity issues. The model fit showed improvement across successive steps; however, according to the Hosmer–Lemeshow test, only Step 4 demonstrated a good model fit (χ2 = 15.44, df = 8, *p* = 0.051), while earlier steps did not meet the criteria for good fit (Step 1: χ2 = 26.36, df = 5, *p* < 0.001; Step 2: χ2 = 34.59, df = 8, *p* < 0.001; Step 3: χ2 = 64.29, df = 8, *p* < 0.001).

**Table 8:** Binary logistic regression analysis of cynicism dimension of burnout

Step	Chi-square (H–L test); (*p*-value)	Variable	B	SE	Wald	df	OR	95% CI
1	26.35 (.000)	Amotivation	0.55	0.10	32.07	1	1.73***	1.43–2.09
		Constant	−2.87	0.54	28.59	1	0.06***	
2	34.60 (.000)	EM – external regulation	0.10	0.03	9.13	1	1.11**	1.04–1.19
		Amotivation	0.54	0.10	29.61	1	1.72***	1.43–2.10
		Constant	−5.01	0.94	28.13	1	.01***	
3	64.29 (.000)	State anxiety	0.04	0.02	7.81	1	1.05**	1.01–1.08
		EM – external regulation	0.10	0.04	8.22	1	1.11**	1.03–1.19
		Amotivation	0.47	0.10	22.13	1	1.60***	1.32–1.95
		Constant	−6.47	1.13	32.82	1	.00***	
4	15.44 (.051)	State anxiety	0.05	0.02	8.22	1	1.05**	1.01–1.08
		IM – achievement	−0.08	0.04	5.17	1	0.92*	0.86–0.99
		EM – external regulation	0.13	0.04	11.184	1	1.14**	1.06–1.24
		Amotivation	0.41	0.10	17.12	1	1.51***	1.24–1.83
		Constant	−5.28	1.23	18.52	1	.01***	

The odds ratios characterizing the relationship between each independent variable and the dependent variable are derived from multiple logistic regression analyses conducted in a stepwise manner

*H–L test* Hosmer and Lemeshow test, *EM* extrinsic motivation, *IM* intrinsic motivation, *OR* odds ratio, *SE* standard error, *df* degree of freedom, *CI* confidence interval

^*^p < .05

^**^p < .01

^***^p < .001

Finally, the relationships of reduced academic efficacy were examined (as depicted in Table 9). In Step 1, achievement (intrinsic motivation) showed a statistically significant association with reduced academic efficacy (B = −0.19, Wald = 30.23, *p* < 0.001). The odds ratio (OR = 0.83, 95% CI = 0.78–0.89) indicates that for each one-unit increase in achievement motivation, the likelihood of reduced academic efficacy decreases by 17%. Then, depression was added in Step 2, which also demonstrated a statistically significant association (B = 0.15, Wald = 17.75, *p* < 0.001), along with achievement (B = −0.19, Wald = 26.88, *p* < 0.001). The odds ratio (OR = 1.17, 95% CI = 1.09–1.25) suggests that for each one-unit increase in depression, the odds of reduced academic efficacy increase by 17%. Step 3 added stimulation (intrinsic motivation) and knowledge (intrinsic motivation), revealing a statistically significant association (B = −0.10, Wald = 4.94, *p* < 0.05), in addition to depression (B = 0.15, Wald = 17.27, *p* < 0.001), and achievement (B = −0.13, Wald = 9.25, *p* < 0.01). Model fit improved progressively across steps (not shown in table). The odds ratio (OR = 0.91, 95% CI = 0.83–0.99) suggests that for each one-unit increase in stimulation, the odds of reduced academic efficacy decrease by 9%. Similarly to the previous two dimensions, for reduced academic efficacy, multicollinearity was not a concern, with all tolerance values exceeding 0.2 and VIF values remaining below the critical level of 5, suggesting that the model’s predictors are not highly correlated. The Hosmer–Lemeshow test described good model fit across all steps (Step 1: χ2 = 8.59, df = 7, *p* = 0.283; Step 2: χ2 = 10.94, df = 8, *p* = 0.205; Step 3: χ2 = 8.51, df = 8, *p* = 0.385).

**Table 9:** Binary logistic regression analysis of reduced academic efficacy dimension of burnout

Step	Chi-square (H–L test); (*p*-value)	Variable	B	SE	Wald	df	OR	95% CI
1	8.59 (.283)	IM – achievement	-.19	0.03	30.23	1	0.83***	0.78–0.89
		Constant	2.06	0.57	13.31	1	7.84***	
2	10.938 (.21)	Depression	0.15	0.04	17.75	1	1.17***	1.09–1.25
		IM – achievement	−0.19	0.04	26.88	1	0.83***	0.77–0.89
		Constant	−0.39	0.79	0.25	1	0.68	
3	8.512 (.39)	Depression	0.15	0.04	17.272	1	1.16***	1.08–1.25
		IM – achievement	−0.13	0.04	9.25	1	0.88**	0.80–0.95
		IM – stimulation	−0.10	0.04	4.94	1	0.91*	0.83–0.99
		Constant	0.24	0.85	0.08	1	1.27	

The odds ratios characterizing the relationship between each independent variable and the dependent variable are derived from multiple logistic regression analyses conducted in a stepwise manner

*H–L test* Hosmer and Lemeshow test, *IM* intrinsic motivation, *OR* odds ratio, *SE* standard error, *df* degree of freedom, *CI* confidence interval

^*^p < .05

^**^p < .01

^***^p < .001

## Discussion

This cross-sectional study aimed to explore the interplay between student burnout and key psychological variables – depression, anxiety, academic motivation, and academic resilience – among medical students. Specifically, the study examined the prevalence of student burnout in a sample of Hungarian medical students, identified differences in burnout dimensions across training stages (preclinical vs. clinical), and assessed the role of specific psychological (risk and protective) factors in relation to student burnout. Within the sample, alarming patterns emerged in the distribution of the independent variables. Most students in this study exhibited mild depressive symptoms, yet even mild depression can significantly impair academic performance and overall well-being [44]. The sample’s mean depression score was notably higher than previously reported in comparable studies [13, 54], emphasizing the need for early monitoring and intervention. Anxiety, another significant risk factor, also emerged as a major challenge, with participants reporting moderate-to-high levels of state anxiety. These levels exceed Hungarian normative scores [39], underscoring the burden of mental health challenges in this population. Addressing anxiety and depression in medical students could therefore serve as a preventive measure to improve well-being and academic performance.

The analysis of academic motivation revealed a diverse distribution, with intrinsic motivation focused on knowledge and extrinsic motivation driven by identified and external regulation being the most prevalent forms. These findings echo the self-determination theory of Deci and Ryan [19], where the alignment of external goals with personal values is seen as a protective factor for well-being and academic success. However, the relatively high levels of introjected regulation and accomplishment-oriented motivation can suggest emotional vulnerability, as these motivations may lead to disengagement from the learning process. It is important to note that comparing these findings with those of other studies on medical students is challenging due to the varying methodologies used [41, 49].

Academic resilience, as a protective factor, showed variability among participants. While they demonstrated strong perseverance and help-seeking tendencies, emotional responses to academic challenges were moderate, suggesting difficulties in managing stress. Mean values of perseverance and willingness to seek help are somewhat lower, however, the mean score for emotional response to academic challenges observed in this study is very similar to that reported in the previous study [15]. These similarities and differences may suggest that while overall academic resilience remains comparable, there are nuances in how it manifests across different student populations.

The dependent variable, student burnout, was defined according to the three dimensions of Schaufeli et al.’s [58] academic burnout model: emotional exhaustion, cynicism, and reduced academic efficacy. The findings reveal that a substantial proportion of students experience burnout, with approximately two-thirds reporting medium or high levels of emotional exhaustion and cynicism (scores above 13.32 and 4.83, respectively – by the Maslach Inventory). These results align with global trends, indicating high burnout rates among medical students [25]. However, it is important to note that these findings are influenced by the dichotomization method used to classify student burnout levels. The dichotomization approach may include students with average burnout scores in the at-risk category, which could lead to an overestimation of the true severity of burnout across the entire sample. As a result, while the study highlights an elevated prevalence of student burnout, it is important to interpret the findings with caution, as the dichotomization may not necessarily reflect an “acute issue” for all students. The elevated prevalence rate in this sample may be partly attributed to the demanding curriculum and intense academic pressure characteristic of the Hungarian medical education system [33]. In addition, limited opportunities for recreation and the lack of structured stress management and resilience-building courses likely contribute to students’ high anxiety and emotional exhaustion, further exacerbating burnout.

Unlike a previous Hungarian study by Győrffy et al. [30], which found no significant variance in burnout dimensions across training stages, this study identified notable differences in emotional exhaustion and cynicism. This divergence may suggest that while emotional exhaustion and cynicism increase with years, the perception of academic efficacy might remain relatively stable, or the effects of different stages of training on academic efficacy could be less pronounced [20, 27]. However, the finding of heightened cynicism among clinical students warrants particular attention, as this aligns with the broader recognition in the literature that idealism at the start of medical training often diminishes over time, replaced by cynicism due to the training process itself [34]. Furthermore, the current study also identifies specific periods where student burnout appears to peak, particularly between the first and fifth years. Contrasting experiences and expectations at these stages of medical training can explain this trend. First-year students are typically highly motivated and eager to learn, with a strong sense of purpose and idealism about their future careers [46]. However, by the fifth year, the reality of the medical profession begins to set in, as students are fully immersed in clinical work. In the Hungarian medical education system, this is the last year with structured lectures and practical sessions before the final year, which is entirely focused on clinical rotations and exams. The pressure to have mastered a vast amount of knowledge by this year, coupled with the looming responsibility of becoming independent physicians within a year, can be anxiety-inducing for many students. Additionally, after five years of intensive study, students may begin to lose some of their initial idealism, as the realities of the medical profession become more apparent, often in great contrast to the idealized portrayals seen in media [11]. Moreover, the fact that the study sample included a higher proportion of female students may have also influenced these results. Research indicates that female medical students often experience heightened tensions due to the simultaneous presence of professional and societal norms, which might have contributed to the significant increase in emotional exhaustion and cynicism observed in the sample [7, 31].

The multiple logistic regression analyses confirmed that burnout dimensions were significantly predicted by depression, anxiety, and various types of motivation; their role might be different depending on each burnout dimension. In terms of student burnout, our research offers a comprehensive view by addressing all burnout dimensions, that is, emotional exhaustion, cynicism, and reduced academic efficacy in a nuanced way. While most research often examines burnout as a single construct [3, 20], our study takes an innovative approach by creating separate predictor models for each of the three burnout dimensions. As a result, comparable data for each burnout dimension are not readily available, highlighting the originality of our study.

In the multivariate analyses, emotional exhaustion was predicted by depression, anxiety, and amotivation. Findings from the literature also suggest that the co-occurrence of depression and anxiety is among the most common mental health problems among medical students globally, further underscoring the heightened vulnerability of this population to burnout [28, 37]. In terms of depressive symptoms, our findings align with previous research, which consistently highlights the role of depression as a critical factor in emotional exhaustion among medical students [2, 60]. Anxiety, although a weaker predictor in this model, can also contribute to emotional exhaustion by exacerbating feelings of being overwhelmed, a phenomenon that has been consistently associated with increased stress and emotional exhaustion in prior research [59]. Amotivation further compounds this by diminishing students’ engagement with their studies, leading to increased exhaustion. These findings align with the results of Zaregar et al.’s study [68], supporting that having lower motivation for continued medical education is indeed linked to increased emotional exhaustion.

Cynicism was predominantly predicted by amotivation and external motivation, with anxiety and achievement-oriented intrinsic motivation also playing significant roles. In this context, amotivation, by triggering a sense of meaninglessness in academic pursuits, can foster a cynical attitude toward studies and the medical profession as a whole, a relationship supported by previous research [68]. The influence of external regulation further contributes to cynicism, as students may feel compelled to study for reasons other than personal growth or interest, which can increase detachment. On the contrary, Pagnin et al. [52] did not find any significant correlations between extrinsic motivations and this burnout dimension. Anxiety, similarly, exacerbates cynicism by creating a constant state of tension. This aligns with previous findings [50, 60], which noted that students who had high levels of anxiety tended to experience increased cynicism. Interestingly, intrinsic motivation, particularly achievement motivation, acted as a protective factor against cynicism. This relationship, where higher cynicism is associated with lower intrinsic motivation, has already been demonstrated in previous studies [55]. This may be explained by the fact that students who are intrinsically motivated by the desire to achieve are less likely to develop cynical attitudes, as they find personal meaning in their academic work. Supporting this idea, Győrffy et al. [30] previously reported that the lack of intrinsic motivation was a major risk factor for cynicism.

Finally, the dimension of reduced academic efficacy was predicted by intrinsic motivation, particularly the achievement component. As can be understood, achievement motivation was not only a protective factor against cynicism, but feelings of inefficacy. Interestingly, another facet of intrinsic motivation, namely stimulation, also played a protective role against reduced academic efficacy, as it reflects pleasure in learning for its own sake. These findings are consistent with previous research showing a significant inverse correlation between motivation and burnout and, more specifically, between motivation and reduced academic efficacy [68]. On the other hand, depression negatively impacted academic efficacy, most probably by undermining self-esteem and creating feelings of incompetence. This aligns with previous data, where low academic efficacy and depressive symptoms were significantly associated [2]. This result is further supported by Lyndon et al. [41], who demonstrated that students with lower intrinsic motivation exhibited lower self-efficacy, which likely contribute to reduced academic efficacy.

### Study strengths and limitations

This research holds significant advantages, as it addresses the global issue of student burnout, which is increasingly observed among medical students, including in Hungary, where data is still limited. A key strength of this study is its use of a well-validated burnout measure, while its focus on both preclinical and clinical stages of medical education provides a comprehensive view of how student burnout evolves throughout medical training. Furthermore, data has been provided on how each of the burnout dimensions fluctuates throughout the medical curriculum, offering deeper insights into the targeted interventions required at each stage. Additionally, this study explores the relationship between academic motivation and student burnout, adding a novel contribution to the field.

However, there are limitations to consider. First, the cross-sectional design limits the ability to infer causality between psychological factors and student burnout, as this design does not allow for the examination of dynamic changes over time or the establishment of causal relationships, meaning the observed associations are merely correlational in nature. Additionally, the decision to combine medium and high-risk burnout groups into a single category may mask important differences between these two groups. Although this approach aligns with previous research [30], further studies could examine these groups separately. Then, the self-reported nature of the data may introduce bias, and the sample, being limited to a single medical school, may affect the generalizability of the findings. The incentive for participation could have influenced response rates and participant responses: students who were motivated by the prospect of earning points may have been more likely to participate and may have overreported negative experiences such as burnout, believing that highlighting challenges would align better with the study’s goals. This could have led to an exaggeration of the prevalence of student burnout, making the findings potentially less reflective of the broader population of Hungarian medical students. Nevertheless, offering this incentive was deemed appropriate to maximize the reach of the study as a pilot research project. There is another risk of selection bias, as students experiencing student burnout may have been more likely to respond to the survey, further impacting the generalizability of the results. Regarding the representativeness of the sample, there were discrepancies between the study sample and the target population in terms of year-wise distribution, with overrepresentation of earlier-year students. This imbalance may limit the generalizability of the findings, particularly regarding burnout experiences in advanced years of medical training. Moreover, the response rates varied across academic years, with Year 1 having the highest response rate and Year 6 showing the lowest. Given that the majority of responses came from students in the earlier stages of their education, the results may not fully represent the experiences of students in later years. Finally, it is important to note that the sample was not selected using a random or stratified sampling method but rather through inclusive distribution of the questionnaire link to all students in the Hungarian medical program. As such, the sample can be considered a convenience sample, which inherently limits its representativeness. This non-random sampling approach may have led to self-selection bias, where students with a greater interest in the topic or those experiencing higher levels of student burnout were more likely to participate. Consequently, the findings may not be fully generalizable to the broader population of Hungarian medical students. Future studies employing stratified or random sampling methods with more balanced participation across academic years could help further explore these differences and provide a clearer understanding of student burnout at various stages of medical training.

### Practical implications

The findings from this study have important implications for medical education systems. To sustain motivation from the early stages of medical training, institutions should implement orientation programs that focus not only on academic preparation but also on stress management, resilience-building, and realistic expectations of the medical profession. Peer mentorship programs could also be beneficial for first-year students with guidance from more experienced students, helping them navigate the initial challenges of medical school. For those in clinical training, medical schools should provide structured support systems such as regular peer support groups for discussing patient interactions and emotional experiences, that is, Bálint groups [67]. These sessions could help students process clinical experiences and manage anxiety. According to the model of Dunn, Iglewicz, and Moutier [22], even though resilience is an individual characteristic, it can be encouraged by the educational institution, for example, by developing individual plans to improve academic performance with study strategies.

Beside year-specific interventions, implications can be organized into personal, organizational, and institutional levels based on the paper of [5]. At the personal level, students should be encouraged to engage in self-care practices and participate in regular stress management workshops. To combat cynicism, students could benefit from reflective practices, such as journaling or peer discussions, helping them reconnect with their initial motivations for pursuing medicine. Building on the study’s results, fostering intrinsic motivation, particularly through setting achievable goals and reinforcing achievement-oriented motivation, can reduce both cynicism and the risk of decreased academic efficacy. Encouraging students to develop effective study habits and seek regular feedback from mentors can further support their academic engagement, as these practices enhance both motivation and a sense of academic accomplishment.

At the organizational level, medical schools should offer structured peer support systems and adjust academic workload to prevent emotional exhaustion. Furthermore, since amotivation can significantly predict emotional exhaustion and cynicism, fostering intrinsic motivation at this level is crucial. This can be achieved by aligning academic tasks with students’ personal interests and ensuring that they feel a sense of meaningfulness in their work. Incorporating elements like problem-based learning and clinical simulations into the curriculum can reduce cynicism and increase engagement. Additionally, providing academic resources like tutoring and courses on emotional intelligence can empower students to manage their emotional well-being more effectively.

On a broader institutional level, it is essential to advocate for policies that create a supportive framework for medical education, including national guidelines for mental health support. Given the findings on the significant role of depression and anxiety in emotional exhaustion, institutions should prioritize mental health resources. National policies should ensure consistent access to mental health support, promoting proactive burnout prevention. Furthermore, ensuring access to high-quality education, including well-trained educators and modern tools, can enhance both academic and personal well-being for students.

## Conclusions

In conclusion, these findings contribute to a deeper understanding of student burnout in the Hungarian medical education context, highlighting both common patterns and some divergences from existing research. The study identifies several risk factors, such as depression, anxiety, and amotivation, which are strongly associated with emotional exhaustion and cynicism, while intrinsic motivation, particularly the achievement and stimulation components, emerged as protective factors against both cynicism and reduced academic efficacy. By identifying differences in student burnout across training stages, interventions can be tailored to reduce burnout within medical schools, potentially leading to a more resilient healthcare workforce. However, due to the cross-sectional design, causal conclusions cannot be drawn, and the potential biases from self-reported data, the sample’s limitation to a single institution, and methodological concerns may affect the generalizability of the results. Future studies would benefit from a longitudinal design and more balanced participation across academic years to further explore the dynamics of student burnout across different stages of medical education.

## Supplementary Information

Additional file 1. “Participants’ demographics” and contains demographic details of the sample.

Additional file 2. “Post-hoc Bonferroni test results for burnout dimensions across academic year groups” and contains all mean differences based on post-hoc tests after MANOVA analysis using Bonferroni correction.

## Data Availability

The datasets used and analyzed during the current study are available from the corresponding author upon reasonable request.

## Abbreviations

AE: Academic efficacy
AMS: Academic Motivation Scale
ARS-30: Academic Resilience Scale
BDI: Beck Depression Inventory
CI: Confidence interval
CY: Cynicism
df: Degree of freedom
EM: Extrinsic motivation
EX: Emotional exhaustion
H–L: Hosmer and Lemeshow test
IM: Intrinsic motivation
MANOVA: Multivariate Analysis of Variance
MBI-SS: Maslach Burnout Inventory Student Survey
OR: Odds ratio
SD: Standard deviation
SE: Standard error
STAI-AD: State-Trait Anxiety Inventory for Adults
US: United States
