# Supplementary material for: Risk and protective factors of student burnout among medical students: a multivariate analysis
Source: BMC Med Educ. 2025 Mar 15;25:386. doi: 10.1186/s12909-025-06956-8 (PMC11910849; doi:10.1186/s12909-025-06956-8)
Supplement: Supplementary file 1 — Additional file 1. “Participants’ demographics” and contains demographic details of the sample. [file 12909_2025_6956_MOESM1_ESM.docx]

**Additional file 1**

*Participants' demographics (N = 214)*

| Variable | n (%) | Mean (SD) Median / range Min–max |
| --- | --- | --- |
| **Biological sex** | | |
| Male | 56 (26.20) |  |
| Female | 158 (73.80) |  |
| **Age** |  | 21.82 (2.63) 21 / 17 18–35 |
| **Academic year** |  |  |
| 1 | 55 (25.70) |  |
| 2 | 45 (21.00) |  |
| 3 | 37 (17.30) |  |
| 4 | 39 (18.20) |  |
| 5 | 23 (10.70) |  |
| 6 | 15 (7.00) |  |
