# Supplementary material for: Risk and protective factors of student burnout among medical students: a multivariate analysis
Source: BMC Med Educ. 2025 Mar 15;25:386. doi: 10.1186/s12909-025-06956-8 (PMC11910849; doi:10.1186/s12909-025-06956-8)
Supplement: Supplementary file 2 — Additional file 2. “Post-hoc Bonferroni test results for burnout dimensions across academic year groups” and contains all mean differences based on post-hoc tests after MANOVA analysis using Bonferroni correction. [file 12909_2025_6956_MOESM2_ESM.docx]

**Additional file 2**

*Post-hoc Bonferroni test results for burnout dimensions across academic year groups*

| Dependent Variable | Academic year comparison | Mean difference | S.E. | p-value | 95% Confidence Interval |
| --- | --- | --- | --- | --- | --- |
| Emotional exhaustion | 1-2 | -3,47 | 1,39 | ,199 | -7,60 to ,66 |
|  | 1-3 | -5,07* | 1,47 | ,010 | -9,43 to -,70 |
|  | 1-4 | -3,75 | 1,45 | ,153 | -8,05 to ,54 |
|  | 1-5 | -7,26* | 1,72 | ,001 | -12,36 to -2,16 |
|  | 1-6 | -4,54 | 2,02 | ,379 | -10,52 to 1,44 |
|  | 2-3 | -1,59 | 1,54 | 1,000 | -6,15 to 2,96 |
|  | 2-4 | -,28 | 1,51 | 1,000 | -4,78 to 4,21 |
|  | 2-5 | -3,78 | 1,77 | ,511 | -9,05 to 1,48 |
|  | 2-6 | -1,07 | 2,06 | 1,000 | -7,19 to 5,06 |
|  | 3-4 | 1,31 | 1,59 | 1,000 | -3,40 to 6,03 |
|  | 3-5 | -2,19 | 1,84 | 1,000 | -7,64 to 3,27 |
|  | 3-6 | ,53 | 2,12 | 1,000 | -5,76 to 6,81 |
|  | 4-5 | -3,50 | 1,82 | ,834 | -8,90 to 1,90 |
|  | 4-6 | -,78 | 2,10 | 1,000 | -7,02 to 5,46 |
|  | 5-6 | 2,72 | 2,30 | 1,000 | -4,10 to 9,53 |
| Cynicism | 1-2 | -4,01* | 1,28 | ,029 | -7,80 to -0,22 |
|  | 1-3 | -4,27* | 1,35 | ,027 | -8,28 to -0,26 |
|  | 1-4 | -5,86* | 1,33 | ,000 | -9,80 to -1,90 |
|  | 1-5 | -11,23* | 1,58 | ,000 | -15,92 to -6,54 |
|  | 1-6 | -8,65* | 1,85 | ,000 | -14,15 to -3,16 |
|  | 2-3 | -,26 | 1,41 | 1,000 | -4,45 to 3,93 |
|  | 2-4 | -1,84 | 1,39 | 1,000 | -5,97 to 2,29 |
|  | 2-5 | -7,22* | 1,63 | ,000 | -12,06 to -2,38 |
|  | 2-6 | -4,64 | 1,90 | ,226 | -10,27 to 0,98 |
|  | 3-4 | -1,58 | 1,46 | 1,000 | -5,91 to 2,75 |
|  | 3-5 | -6,96* | 1,69 | ,001 | -11,97 to -1,95 |
|  | 3-6 | -4,38 | 1,95 | ,380 | -10,16 to 1,39 |
|  | 4-5 | -5,39* | 1,67 | ,022 | -10,34 to -0,42 |
|  | 4-6 | -2,81 | 1,93 | 1,000 | -8,54 to 2,93 |
|  | 5-6 | 2,57 | 2,11 | 1,000 | -3,69 to 8,84 |
| Reduced academic efficacy | 1-2 | 1,58 | 1,22 | 1,000 | -2,03 to 5,20 |
|  | 1-3 | 1,94 | 1,29 | 1,000 | -1,89 to 5,76 |
|  | 1-4 | 1,74 | 1,27 | 1,000 | -2,02 to 5,50 |
|  | 1-5 | ,68 | 1,50 | 1,000 | -3,78 to 5,15 |
|  | 1-6 | 3,27 | 1,76 | ,974 | -1,96 to 8,51 |
|  | 2-3 | ,35 | 1,34 | 1,000 | -3,64 to 4,34 |
|  | 2-4 | ,16 | 1,33 | 1,000 | -3,78 to 4,09 |
|  | 2-5 | -,90 | 1,55 | 1,000 | -5,51 to 3,71 |
|  | 2-6 | 1,69 | 1,81 | 1,000 | -3,67 to 7,05 |
|  | 3-4 | -,20 | 1,39 | 1,000 | -4,32 to 3,93 |
|  | 3-5 | -1,26 | 1,61 | 1,000 | -6,03 to 3,52 |
|  | 3-6 | 1,34 | 1,85 | 1,000 | -4,17 to 6,84 |
|  | 4-5 | -1,06 | 1,59 | 1,000 | -5,79 to 3,67 |
|  | 4-6 | 1,53 | 1,84 | 1,000 | -3,93 to 7,00 |
|  | 5-6 | 2,59 | 2,01 | 1,000 | -3,38 to 8,56 |

*Note:* The table displays all mean differences based on post-hoc tests after MANOVA analysis using Bonferroni correction.

* The mean difference is significant at the 0.05 level.
